# Electrocardiographic Abnormalities and QT_c_ Interval in Patients Undergoing Hemodialysis

**DOI:** 10.1371/journal.pone.0155445

**Published:** 2016-05-12

**Authors:** Yuxin Nie, Jianzhou Zou, Yixiu Liang, Bo Shen, Zhonghua Liu, Xuesen Cao, Xiaohong Chen, Xiaoqiang Ding

**Affiliations:** 1 Division of Nephrology, Zhongshan Hospital, Fudan University, Shanghai, P. R. China; 2 Shanghai Institute of Kidney Disease and Dialysis, Shanghai, P. R. China; 3 Key Laboratory of Kidney and Blood Purification of Shanghai, Shanghai, P. R. China; 4 Division of Cardiology, Zhongshan Hospital, Fudan University, Shanghai, P. R. China; University of Tampere, FINLAND

## Abstract

**Background:**

Sudden cardiac death is one of the primary causes of mortality in chronic hemodialysis (HD) patients. Prolonged QT_c_ interval is associated with increased rate of sudden cardiac death. The aim of this article is to assess the abnormalities found in electrocardiograms (ECGs), and to explore factors that can influence the QT_c_ interval.

**Methods:**

A total of 141 conventional HD patients were enrolled in this study. ECG tests were conducted on each patient before a single dialysis session and 15 minutes before the end of dialysis session (at peak stress). Echocardiography tests were conducted before dialysis session began. Blood samples were drawn by phlebotomy immediately before and after the dialysis session.

**Results:**

Before dialysis, 93.62% of the patients were in sinus rhythm, and approximately 65% of the patients showed a prolonged QT_c_ interval (i.e., a QT_c_ interval above 440 ms in males and above 460ms in females). A comparison of ECG parameters before dialysis and at peak stress showed increases in heart rate (77.45±11.92 vs. 80.38±14.65 bpm, *p* = 0.001) and QT_c_ interval (460.05±24.53 ms vs. 470.93±24.92 ms, *p*<0.001). After dividing patients into two groups according to the QT_c_ interval, lower pre-dialysis serum concentrations of potassium (K^+^), calcium (Ca^2+^), phosphorus, calcium* phosphorus (Ca*P), and higher concentrations of plasma brain natriuretic peptide (BNP) were found in the group with prolonged QT_c_ intervals. Patients in this group also had a larger left atrial diameter (LAD) and a thicker interventricular septum, and they tended to be older than patients in the other group. Then patients were divided into two groups according to ΔQT_c_ (ΔQT_c_ = QT_c peak-stress_- QT_c pre-HD_). When analyzing the patients whose QT_c_ intervals were longer at peak stress than before HD, we found that they had higher concentrations of Ca^2+^ and P^5+^ and lower concentrations of K^+^, ferritin, UA, and BNP. They were also more likely to be female. In addition, more cardiac construction abnormalities were found in this group. In multiple regression analyses, serum Ca^2+^ concentration before HD and LAD were independent variables of QT_c_ interval prolongation. UA, ferritin, and interventricular septum were independent variables of ΔQT_c_.

**Conclusion:**

Prolonged QT interval is very common in HD patients and is associated with several risk factors. An appropriate concentration of dialysate electrolytes should be chosen depending on patients’ clinical conditions.

## Introduction

Patients with end-stage renal disease (ESRD) undergoing hemodialysis (HD) have a high prevalence of electrocardiograms (ECG) abnormalities [1] and an elevated mortality rate in comparison with that of the general population. In about 30% of the patients, death correlated with the occurrence of cardiovascular disorders such as arrhythmias and sudden cardiac arrest [2–3]. The increased mortality may be due not only to the presence of the traditional risks such as coronary disease, left ventricular hypertrophy, and diabetes. It may also be an effect of stress caused by electrolyte, acid-base balance, and plasma volume changes associated specifically with HD treatment on a heart that is already in an unhealthy state [4].

Changes in heart electrical systole during dialysis can provide essential information on cardiac electrical activities, and can predict potentially harmful arrhythmias. ECG tests can be administered in a simple, non-invasive, and inexpensive way, and ECG changes are frequently found in individuals undergoing HD. ECG abnormalities, especially prolonged QT interval, may increase the risk of sudden cardiac death [5–6]. Vazquez and his colleagues found that patients who had a myocardial infarction and ECG abnormalities at the start of dialysis were at a 7-fold greater risk of sudden death than those did not have these risk factors [7]. Some studies have demonstrated that the electrolytes of the dialysate and a patient’s nutrition status and heart function are closely associated with abnormal QT intervals [2,5,8]. However, no consensus has been reached yet. Also, the sample sizes of previous studies are limited.

This study was undertaken to assess the ECG abnormalities in maintenance hemodialysis (MHD) patients and changes in the ECG during a single HD session. We also wished to explore risk factors for QT interval prolongation.

## Patients and Methods

Patients undergoing HD for more than 3 months were invited to participate in this study, conducted from March to May 2014 at the Blood Purification Center of Fudan University, Zhongshan Hospital. The exclusion criteria were participation in a single session of HD lasting up to 3 hours, congenital long QT syndrome, a pacemaker, cardiac resynchronization therapy, an implantable cardioverter defibrillator, or taking drugs that might have an effect on QT interval. All patients were undergoing a standard 4-hour dialysis 3 times a week. Bicarbonate dialysate containing 2.0 mmol/L K^+^, 1.25 mmol/L calcium (Ca^2+^), 138mmol/L sodium (Na^+^), and 0.5 mmol/L magnesium (Mg^2+^) was used.

We conducted ECG tests on each patient 10 minutes before HD and 15 minutes before the end of HD (at peak stress [9]). The recordings were taken using a 12-lead machine (MAC 1200, GE) at 10mm/mv and 25mm/s. QT interval was measured for each ECG. The QT interval was defined as the time between the start of the Q wave and the end of the T wave, and it was measured by an investigator who was blinded to patients’ clinical and laboratory results of patients manually. Leads without U waves were our first choice [10]. A tangent line was drawn to the steepest slope of the T wave, and the intersection of the tangent line and the baseline was considered to be the end of the T wave. If there was no lead without U waves, then the measurement depended on the morphology of the U wave. U waves separated by T waves were excluded, while larger U waves fused to T waves were measured by a tangent line drawn to the steepest slope of the last limb of the T wave [11]. The corrected QT interval (QT_c_) was estimated by Bazett’s formula (QT_c_ = QT/√RR, RR = 60/HR) [8]. A prolonged QT_c_ interval was defined as greater than 440 ms in males and greater than 460ms in females.

In addition to QT interval, heart rate, rhythm, and conduction abnormalities were recorded on each ECG. The following criteria were used to diagnose of ECG abnormalities. (1) A first-degree atrioventricular block (AVB) was defined as a prolongation of the PR interval above the normal range. A second-degree AVB was defined as both a gradual increase of the PR interval until a P wave is lost (Mobitz 1) and consecutively conducted beats with the same PR interval followed by a dropped P wave (Mobitz 2). If ECG shows that QRS waves were conducted at their own rate and totally independent of the P waves, it was defined as a third-degree AVB. All three degrees of AVB were called AVBs. (2) A QRS wave originating from a supraventricular electrical activity with a duration equals to or greater than 120ms was defined as bundle branch block. If there was a tall, broad R wave in the I and the V _6_ lead, and a QS or rS in the V_1_ lead, it was a diagnosed as left bundle branch block (LBBB). If there was an rsR’ wave or a tall, broad R wave in the V_1_ lead, and a wide, slurred S wave in the I and the V_5-6_ leads, it was diagnosed as right bundle branch block (RBBB). (3) Left ventricular hypertrophy was determined by the Sokolow-Lyon criteria (SV_1_+RV_5_/RV_6_>35mm) [12].

Two-dimensional echocardiography was conducted on each patient before the HD session (GE medical systems, Germany) by a single doctor, who was blinded to other patient data. Information on basic cardiac structures, such as LAD, interventricular septum (IVS), left ventricular posterior wall thickness (LVPW), left ventricular end-systolic diameter (LVESD), left ventricular end-dialytic diameter (LVEDD), and valve calcifications was recorded. The left ventricular ejection fraction (LVEF) was estimated using a biplane method.

Venous blood samples were drawn by phlebotomy immediately before and after each dialysis session. All biochemical analyses including serum albumin, pre-albumin, hemoglobin, serum creatinine (SCr), blood urea nitrogen (BUN), UA, Na^+^, K^+^, Ca^2+^, P^5+^, Mg^2+^, and ferritin were measured using an automatic analyzer in clinical laboratories. The concentration of high-sensitivity C-reactive protein (hsCRP) was determined using immunoturbidimetry assay. N-terminal proBNP was assessed using enzyme-linked immunosorbent assay (ELISA).

The study was conducted according to the principles expressed in the Declaration of Helsinki. Study proposal was approved by the Ethical Committee of Zhongshan Hospital, Fudan University. During data collection, all information was recorded using a database from which patient identification information had been removed.

### Statistical Analyses

SPSS software package (version 20.0) was used for statistical analysis. Statistical significance level was defined as 0.05. Data were expressed as counts/percentages for discrete variables or as means ± SDs for continuous variables. A comparison between patients with and without QT_c_ interval prolongation was made by a chi-square test for categorical variables. For normally distributed continuous variables and non-normally distributed continuous variables, we chose a *t* test or a Mann-Whitney test, respectively. The Pearson correlation was used to assess linear relationships. Univariate and multivariate logistic regression analyses were applied to identify risk factors for QT_c_ interval prolongation. Multivariate analysis involved variables showed statistical differences in univariate analysis.

## Results

A total of 141 patients were invited to participate in this study conducted from March to May 2014 at the Blood Purification Center of Zhongshan Hospital, Fudan University. Among study participants, 108 (76.6%) were male and 33 (23.4%) were female. The mean age was 60.9 years, and the mean duration of HD was 34.3 months. Among these patients, 46.8% of them used an artery-vein fistula (AVF) as the vascular access. The baseline characteristics are shown in Table 1.

**Table 1:** Baseline characteristics of the study patients.

Characteristics	Patients
Number of patients	141
Male/Female	108/33
Mean age (yr)	60.87±15.73
Age when start chronic HD (±SD, yr)	58.01±15.73
Mean duration of chronic HD (±SD, month)	34.34±16.26
Vascular access, AVF/Catheter	66/75
Diabetes (Y/N)	18/123
Hypertension (Y/N)	70/71
LVH (Y/N) ^a^	62/79

^a^ LVH = Left Ventricular Hypertrophy. LVH was determined based on ECG results using the Sokolow-Lyon criteria.

According to our analysis of the ECGs before HD, 132 patients (93.62%) were in sinus rhythm. Electrical conduction disturbances (including AVB, LBBB, and RBBB were found in 39 patients (27.66%). Approximately 65% of the patients showed prolonged QT_c_ intervals. QT_c_ intervals ranged from 389 ms to 510 ms in the group overall. The abnormal ECG findings are listed in Table 2.

**Table 2:** Electrocardiographic variables in patients undergoing HD (n = 141).

Abnormal electrocardiographic findings	HD patients (n/%)
Atrial fibrillation	9/6.38%
Atrial premature beats	6/4.26%
Ventricular premature beats	13/9.22%
AVB^a^	20/14.18%
LBBB^b^	3/2.13%
RBBB^c^	16/11.35%
Prolonged QT_c_ interval	86/65.15%

^a^ AVB = Atrioventricular block

^b^ LBBB = Left bundle branch block

^c^ RBBB = Right bundle branch block

Since the QT interval is hard to measure under a fibrillation rhythm, and varying of the RR interval may result in variation of the QT_c_ interval [10, 13], we excluded the 9 patients with atrial fibrillation. Then we compared the remaining 132 patients’ pre-dialysis ECGs with their peak stress ECGs. Significant differences were found between these two time points. At peak stress, the heart rate increased significantly from 77.45±11.92 beats per minutes (bpm) to 80.38±14.65 bpm (*p* = 0.001), while the PR duration decreased from 173.55±30.26 to 169.11±31.30 ms (*p*<0.001). The width of the QRS wave increased from 99.20±15.11 to 100.55±15.25 ms (*p* = 0.007). The maximum QT_c_ interval was significantly prolonged at peak stress during HD (460.05±24.53 ms vs. 470.93±24.92 ms, *p*<0.001). All of these findings are indicative of disturbed cardiac electrical activity induced by HD. Differences of measured variables before and at peak stress during HD are shown in Table 3.

**Table 3:** Electrocardiogram variables in different time points in patients undergoing HD (n = 132).

Variable	Pre-HD	Peak Stress	t	p value
Heart rate (bpm)	77.45±11.92	80.38±14.65	-3.413	0.001
P wave (ms)	112.3±11.68	113.59±11.11	-1.259	0.210
PR (ms)	173.55±30.26	169.11±31.30	4.449	<0.001
QRS wave (ms)	99.20±15.11	100.55±15.25	-2.745	0.007
QT_max_ (ms)	407.91±31.53	411.12±35.19	-1.777	0.078
QT_cmax_ (ms)	460.05±24.53	470.93±24.92	-6.290	<0.001

Among the sinus rhythm patients, 24 were excluded because they were unwilling to allow us to take blood samples both before and after HD. The remaining 108 patients were classified into two groups based on QT_c_ intervals. As shown in Table 4, notable differences were found between the 2 groups including pre-HD serum concentrations of potassium, calcium, phosphorus, and calcium* phosphorus (Ca*P). The QT_c_ interval was more prone to prolongation in patients who were older when they underwent this study and when they started HD. Moreover, patients with prolonged QT_c_ intervals presented with higher Log BNP levels compared to patients whose QT_c_ interval was normal. A prolonged QT_c_ interval was also more common in patients with a larger LAD and a thicker IVS. No differences were associated with gender, diabetes, vascular access, dialysis vintage, interdialytic weight gain (IDWG), ultra-filtration volume, blood pressure reduction, baseline LVEF, or valve calcification.

**Table 4:** Comparative analysis of variables according to the QT_c_ interval in patients undergoing HD (n = 108).

Variable	QTc ≤ 440ms (if male) QTc ≤ 460ms (if female) (n = 35)	QTc > 440ms (if male) QTc > 460ms (if female) (n = 73)	*p* value
Age (y)	52.94±16.23	64.25±15.50	0.001
Age when start HD (yr)	49.91±16.25	61.63±15.45	<0.001
Pre-HD serum K (mmol/L)	5.09±0.70	4.54±0.80	0.001
Pre-HD serum Ca (mmol/L)	2.44±0.19	2.32±0.16	0.001
Pre-HD serum P (mmol/L)	2.44±0.60	1.99±0.77	0.003
Pre-HD serum Ca*P	6.01±1.70	4.64±1.95	0.001
Pre-HD Log BNP (pg/ml)	3.65±0.40	3.85±0.52	0.034
LAD (mm)	37.80±4.93	41.26±3.47	0.001
IVS (mm)	10.60±1.38	11.66±1.93	0.005

Comparing ECGs before HD and at peak stress, we classified patients into two groups according to ΔQT_c_ (ΔQT_c_ = QT_c peak-stress_- QT_c pre-HD_). As shown in Table 5, significant differences in electrolytes were found between the 2 groups. Analyzing the patients whose QT_c_ interval got longer at peak stress than before HD, we found they had higher levels of ferritin, uric acid, and Log BNP, and that they were more likely to be female. They also had some cardiac construction abnormalities: The LAD, IVS, and LVPW were significantly larger in these patients than in patients whose ΔQT_c_ was less than or equal to 0.

**Table 5:** Comparative analysis of variables according to the QT_c_ change between pre-HD and peak stress (n = 108).

Variable	ΔQT_c_≤0 (n = 34)	ΔQT_c_>0 (n = 74)	*p* value
Gender M/F	31/3	53/21	0.023
Pre-dialysis serum Ca (mmol/L)	2.30±0.20	2.38±0.17	0.043
Pre-dialysis serum P (mmol/L)	1.93±0.71	2.24±0.75	0.045
Pre-dialysis serum Ca*P	4.50±1.69	5.38±2.03	0.022
ΔK (mmol/L) ^a^	-1.15±0.64	-1.39±0.50	0.040
Ferritin (ng/ml)	154.19±117.08	322.75±232.53	<0.001
Pre-dialysis UA (μmol/L)	390.00±54.46	446.23±77.33	<0.001
Pre-dialysis Log BNP (pg/ml)	3.64±0.45	4.10±0.43	<0.001
LAD (mm)	39.04±4.39	42.32±3.22	<0.001
IVS (mm)	10.97±1.50	12.06±2.17	0.011
LVPW (mm)	10.32±1.40	11.53±1.40	<0.001

^a^ ΔK = K _peak-stress_- K _pre-HD_

In multiple regression analyses, calcium before HD, and LAD were independent variables of QT_c_ interval prolongation. UA, ferritin, and IVS were independent variables of ΔQT_c_.

## Discussion

Cardiovascular disease is the main cause of death in MHD patients, which is much higher than in normal people. It usually occurs suddenly, not only because of the high prevalence of traditional risk factors such as hypertension, diabetes, and ischemia myopathy, but also for reasons that remain unclear. When comparing the risk of fatal arrhythmia, it is higher in HD patients than in peritoneal dialysis (PD) patients (62‰ vs. 42‰) [14]. It appears that HD itself is a risk factor for sudden cardiac death due to hemodynamic overload and inflammatory stress. Some researchers have found various types of arrhythmia among HD patients [1]. In our study, a majority of the patients presented in sinus rhythm while 9 patients (6.38%) presented with atrial fibrillation, and nearly 30% of the patients presented with electrical conduction disturbance, similar to HD patients in other studies [15–16].

Rates of sudden death among dialysis patients reportedly range from 4.5 to 12 per 100,000 dialysis sessions [17–19]. In the German Diabetes and Dialysis Study (the 4D Study), 160 of the 1,255 patients (13%) experienced sudden death during the 4-year follow-up [20]. Quite a lot of evidence shows that QT interval is closely related to ventricular action and is reported to predict occurrences of fatal arrhythmias [21–22]. In our study, approximately 65% of the patients had a prolonged QT_c_ interval, which was midway between the rates found by Bignotto et al [1], Sherif et al [10], and Alabd et al [23].

When comparing ECGs before HD and at peak stress, we found great differences in heart rate, PR duration, QRS wave, and QT_c_ interval. The ECG is representative of cardiac electrical activities, and the changes noted at the different time points reflect the unstable status of electrophysiology during the dialysis session. In our study, the heart rate increased from 77.45±11.92 bpm at baseline to 80.38±14.65 bpm at peak stress. The changes in heart rate might be a compensation mechanism in response to fluid removal, electrolyte and pH changes, or HD-induced myocardial ischemia/stunning. Though the heart rate at peak stress is still within normal limits, and the association between the change of heart rate and long-term survival is not yet clear, investigators have found that lower heart rate variability during HD is a predictor for cardiovascular events and death [24]. It has also been shown that a higher heart rate is closely related to lower heart rate variability, even when the heart rate is still within the normal range [25]. Thus, further studies that include heart rate variability would be helpful. Beyond that, studies that use continuous monitoring such as Holter monitoring instead of ECG recordings at specific time points should be considered. There was also a slight increase of QRS duration at peak stress during HD, in agreement with the results of Salari et al [26]. Evidence has shown that in both ischemia cardiomyopathy patients and non-ischemia cardiomyopathy patients, abnormal intraventricular conduction is a risk factor for mortality and sudden death [27–28]. However, similar to heart rate, the QRS duration at peak stress in our study was still within the normal range. One possible explanation is that the changes in heart rate and QRS duration are correlated with changes of serum concentrations of electrolytes [29–30]. So in patients with more severe electrolytes disorders, the changes in heart rate and QRS duration could be more significant and possibly exceed the upper limit of normal. At the same time, although we didn’t exclude patients with chronic heart failure when we started this study, our baseline LVEFs were all above 60%, our patients exhibited normal cardiac function and were perhaps more tolerant of the stress caused by the volume, acid-base and electrolytes changes during HD sessions than patients with cardiac dysfunction. These ECG parameters might change more significantly in patients with basic cardiac diseases. The meaning of the slight increase of QRS duration should be further explored. The QT interval reflects the repolarization of ventricle. More than three-quarters of the patients in our study were observed to have a prolonged QT_c_ interval. Moreover, when at peak stress, the QT_c_ was significantly longer than at baseline. These results are in accordance with other studies [19, 22]. In one of these studies, 47 patients underwent ECG tests before, during, and after a HD session. The maximum QT_c_ interval and QT_c_ dispersion increased after dialysis, and the difference was significant for both [31].

There is a tendency for older patients (no matter when they enrolled in a study or when dialysis started) to present with a longer QT_c_ interval. Mangoni et al [32] found that age independently predicted QT interval in healthy subjects. Reardon and Malik [33] also found that a prolonged QT interval is correlated with increasing age and may be one cause of the increased rate of ventricular arrhythmias and cardiac death in elderly patients. This phenomenon might be due to disturbance of the autonomic nervous system or secondary to age-related cardiac hypertrophy and myocardial action potential prolongation.

Compared with the group whose QT_c_ interval was in the normal range, the prolonged QT_c_ interval group had lower plasma concentrations of K^+^, Ca^2+^, and P^5+^ in our study. When considering QT_c_ interval changes during the HD session, we found a negative correlation between QT_c_ changes and Ca^2+^ and P^5+^ concentrations and K^+^ reduction. Sherif et al [10] found that each mmol/L increase of serum K^+^ concentration may result in a 16ms reduction of the QT_c_ interval. Alabd et al [23] reported a negative correlation between the decrease of serum potassium and the change of QT_c_ interval duration before and after dialysis: The more the serum potassium decreased, the longer the QT_c_ interval post dialysis. These results are similar to the findings in our study. Genovesi et al [34] found that QT_c_ interval was negatively correlated to Ca^2+^ and K^+^ plasma concentration changes. Moreover, when they used dialysates with various concentrations of electrolytes (K^+^ of 2/3 mmol/L; Ca^2+^ of 1.25/1.5/1.75 mmol/L), they found that compared to patients who use dialysate with higher concentrations of K^+^ and Ca^2+^, those who use dialysate with lower concentrations of K^+^ and Ca^2+^ were more likely to have QT_c_ intervals greater than 440 ms. Di Iorio et al [35] also found that patients using dialysate with the lowest concentrations of Ca^2+^ and K^+^ and the highest concentrations of HCO_3_^-^ are the most likely to show prolonged QT_c_ intervals. Genovesi et al [34] stated that prolongation of the QT interval during HD sessions may increase the risk of fatal arrhythmia. Kim ED and Parekh RS [36] reviewed 15 studies on the association of Ca^2+^ with arrhythmias in dialysis and found that 12 studies indicated varying degrees of correlation between serum or dialysate Ca^2+^ and QT_c_ interval or QT dispersion. Low concentrations of serum Ca^2+^ and dialysate Ca^2+^ and rapid reduction of serum Ca^2+^ may lead to an elevated risk of QT_c_ interval prolongation. Therefore, to minimize QT_c_ interval and QT_c_ interval changes related to electrolyte concentrations, higher levels of K^+^, Ca^2+^, and P^5+^ are preferred. Moreover, according to other studies mentioned previously, dialysate with higher concentration of K^+^ and Ca^2+^ should be considered. However, according to clinical practice, dialysate with high Ca^2+^ concentration should be avoided to reduce risk of hypercalcemia and ectopic calcifications. Moreover, since hyperkalemia is high prevalent in HD patients before the beginning of HD session, dialysate with high K^+^ concentration may not be capable of reducing plasma K^+^ sufficiently [32]. High plasma P^5+^ levels are also associated with increased risk of renal osteodystrophy. Because of these contradictions, the electrolyte concentrations of both plasma and dialysate should depend on patients’ clinical conditions. For instance, for patients without hyperkalemia and hypercalcemia, physicians could choose a dialysate with higher concentrations of potassium and calcium to maintain an appropriate level of serum electrolytes and avoid too much change after a single dialysis session. Considering the risk of electrolyte disturbance and calcification, regular assessments are quite necessary. The risk of QT_c_ interval over the upper normal limits should be weighed against the risk of other possible complications.

In patients undergoing conventional HD, cardiac structural and functional abnormalities can affect ventricular repolarization and may contribute to the high incidence of cardiac arrhythmias [37]. In our study, we found that patients whose QT_c_ intervals were longer at peak stress than before dialysis had thicker IVS and LVPW, and a larger left atrial diameter. Medenwald et al [38] analyzed data from the CARLA study and concluded that in the general population, the association between QT_c_ and general mortality is strongest in subjects with increased diastolic thickness of the left ventricular posterior wall. In HD patients, Bignotto et al [1] found that patients with prolonged QT_c_ intervals were more likely to have left ventricular hypertrophy (LVH) comparing to those with normal QT_c_ intervals, defining LVH according to ECGs. Another study [39] dealt with patients suffering prolonged QT_c_ intervals accompanied by ECG-LVH (HR 1.83, 95% CI 1.31–2.57) and at high risk of stroke. But when compared with patients with prolonged QT_c_ intervals without ECG-LVH, this group of patients had an even higher risk (HR 2.70, 95% CI 1.48–4.94) of stroke. This suggests that QT_c_ interval prolongation is more dangerous when it is not secondary to cardiac structural abnormality.

In our study, patients with prolonged QT_c_ intervals had higher levels of serum BNP than those with normal QT_c_ intervals. Moreover, when we compared QT_c_ intervals before HD and at peak stress, we found that patients with a higher concentration of serum BNP had a higher tendency of HD-induced QT_c_ interval prolongation. These results are supported by other research [40].In a study of 398 patients who had experienced heart failure and were followed up for one year [41], investigators found that a BNP increase was associated with higher risk of SCD only in patients with prolonged QT_c_ intervals. Patients with prolonged QT_c_ intervals were under a 3 times greater risk of sudden cardiac death than those without QT_c_ interval prolongation during the one year follow up. Research on the relationship between QT_c_ interval, BNP, and prognostics in HD patients is limited; further exploration is needed.

Patients with the specific characteristics that we mentioned before are more likely to have a greater arrhythmic predisposition during dialysis sessions, and this might help us predict and explain the sudden death caused by arrhythmia in individuals with ESRD undergoing HD.

There are some limitations to our study. We conducted ECG tests only at the beginning and at peak stress during a HD session and found significant differences between these two time points. But our results do not include ECG parameters assessed during the recovery period after HD. Our results could be better illustrated if we had gathered and compared data from the whole HD session. Moreover, we used a surface ECG to assess ventricular electrical activity, which is transient. A continuous monitor would be better. Some studies have shown that QT_c_ interval dispersion might be influenced by HD and could be a predictor of CV events or mortality. However, because of the lack of repeatability, we did not choose that as a parameter in our study.

## Conclusion

Prolonged QT interval is very common in HD patients and is worth close attention. Lower serum K^+^, Ca^2+^ and dialysate Ca^2+^ may lead to higher risk of QT_c_ prolongation. So an appropriate concentration of dialysate electrolytes should be chosen depending on patients’ clinical conditions.

## Supporting Information

S1 File Ethic. Approval Letter of Ethics Committee.
(PDF)

S2 File Checklist. PLOSOne_Clinical_Studies_Checklist.
(DOCX)

S3 File Dataset. Raw data.
(XLSX)
