# Supplementary material for: Electrocardiographic Abnormalities and QTc Interval in Patients Undergoing Hemodialysis
Source: PLoS One. 2016 May 12;11(5):e0155445. doi: 10.1371/journal.pone.0155445 (PMC4865146; doi:10.1371/journal.pone.0155445)
Supplement: S1 File — Approval Letter of Ethics Committee. (PDF) [file pone.0155445.s001.pdf]

伦理委员会批准函
Approval Letter of Ethics Committee

审查编号 Approval No.:**B2013-139**　　**审查日期** Date of Review:2013 年 12 月 26 日

**项目名称** Study Title	维持性血液透析患者心电图异常情况及 QT 间期改变<br>Electrocardiographic abnormalities and QTc interval in patients undergoing hemodialysis				
**试验产品名称** Study Product Name	NA	**产品类别/型号** Product Category	NA	**研究分期** Phase of Study	NA
**批准文号及发文单位**(Approval No. and Issued By):<br>NA			**药检报告及批号**(Certificate of Analysis and Batch No.): NA		
**主要研究者**(Principal Investigator):<br>丁小强			**申办者**(Sponsor):<br>复旦大学附属中山医院		
**审查方式 (Type of Review)**	■会议审查 **(Meeting Review)**　□快速审查**(Expedited Review)**				
**会议地点 (Meeting Location)**	复旦大学附属中山医院 5 号楼 507 会议室				
**会议出席情况 (Meeting Attendance)**	出席**(Attendance)**8人，投票**(Vote)**8人，回避**(Avoidance)**0人（　）				

下面划[√]的研究相关文件已经审阅

The following items[√] have been reviewed in connection with the above study to be conducted by the above investigator

[√] 研究方案及日期 Protocol No.,dated and version: v1.0,2013.11.13

[√] 受试者知情同意书及日期 Consent Form(s) dated and version: v1.0,2013.11.13

[ ] 受试者招募广告及日期 Advertisements for Recruitment dated:

[√] 其他(请具体列出) Other(specify):主要研究者简历;参加人员名单。

审查决定 Decision for this proposal and have been [√]:

[√] 同意 Approval

持续审查频率： □3 个月/3 months　□6 个月/6 months　[√]1 年/1year　□不适用/NA

主任委员/副主任委员签名：[signature]

批准日期：2013,12,30

复旦大学附属中山医院伦理委员会（盖章）

[Stamp: 复旦大学附属中山医院 医学伦理委员会]

注：本批件有效期为一年，逾期未实施的，则自行废止。

联系方式：上海市枫林路 180 号，黄锦培/杨梦婕，电话/传真：：64041990-3257

声明

1. 复旦大学附属中山医院伦理委员会（以下简称本伦理委员会）的职责、人员组成、操作规范和记录遵循 ICH-GCP 及中华人民共和国食品药品监督管理局颁布的《药品临床试验质量管理规范（GCP)》和《药物临床试验伦理审查工作指导原则》，并遵守中国相关法律和法规的规定。
2. 研究应遵循已经由伦理委员会批准的方案执行，应符合赫尔辛基宣言和 CFDA/GCP 的原则。
3. 本批件可能在其他中心机构及其伦理审查委员会备案。如果对方案在贵机构的可行性（包括研究者的资格与经验、设备与条件等）有不同意见，请及时与本伦理委员会联系。
4. 研究过程中，对研究方案和知情同意书等相关文件所作的任何修改，均需得到伦理委员会审查同意后方可实施
5. 在复旦大学附属中山医院发生的严重不良事件或严重非预期不良事件需在向 CFDA 上报的同时递交伦理委员会，国内其他中心发生的严重不良事件或严重非预期不良事件需每月汇总后递交伦理委员会，对于国外发生的严重非预期不良事件每个月汇总后递交伦理委员会，伦理委员会有权对其评估做出新的决定。
6. 本伦理委员会按照国家有关规定，对研究项目进行跟踪审查，自伦理委员会审查意见批复单批准之日起，请研究者在规定的持续审查日期到期或伦理委员会审查意见批复单失效前 1 个月递交持续审查申请，以获得伦理委员会的批准。
7. 暂停/提前终止临床试验，需及时通知本伦理委员会。
8. 方案违背和偏离需及时报告本伦理委员会。
9. 研究结束时，请向本伦理委员会递交结题报告和分中心小结表。
10. 研究中涉及采集生物标本的项目必须遵循本院的《生物标本应用暂行管理办法》，并在获得伦理批准后填写《生物标本应用申请表》。

注：本批件有效期为一年，逾期未实施的，则自行废止。

联系方式：上海市枫林路 180 号，黄锦培/杨梦婕，电话/传真：：64041990-3257

A-024

复旦大学附属中山医院
伦理委员会委员名单及出席情况

ETHICS COMMITTEE COMPOSITION AND ATTENDING

会议编号：2013 年第 12 次会议　　审查日期：Date of Review：2013 年 12 月 26 日

委员姓名和职称 Member Name and Title		职业 Occupation (position)	性别 Male/ Female	工作单位 Working Place	出席者签名 Signature of Attending
姓名	职称/职务				
王玉琦** Wang Yuqi	教授 Professor	医师 Surgeon	男 male	中山医院 Zhong Shan Hospital	
秦新裕* Qin Xinyu	教授 Professor	医师 Surgeon	男 male	中山医院 Zhong Shan Hospital	
樊嘉* Fan Jia	教授 Professor	医师 Surgeon	男 male	中山医院 Zhong Shan Hospital	
王吉耀 Wang Jiyao	教授 Professor	医师 Physician	女 female	中山医院 Zhong Shan Hospital	
朱同玉 Zhu Tongyu	教授 Professor	医师 Surgeon	男 male	中山医院 Zhong Shan Hospital	
刘康达 Liu Kangda	研究员 Researcher	医师 Physician	男 male	中山医院 Zhong Shan Hospital	
李海青 Li Haiqing	副科长 Vice Section Chief	职员 Clerk	男 male	中山医院 Zhong Shan Hospital	
衡慧珠 Heng Huizhu	居委会主任 Residents' Committee	群众代表 Layperson	女 female	枫林街道 Fenglin Community	
李雪宁 Li Xuening	主任药师 Chief Pharmacist	药师 Pharmacist	女 female	中山医院 Zhong Shan Hospital	
张博恒 Zhang Boheng	主任医师 Chief Physician	医师 Physician	男 male	中山医院 Zhong Shan Hospital	
孙元 Sun Yuan	委员 Member	律师 Lawyer	男 male	上海管博律师事务所 Shanghai Guanbo Law Firm	

注：** 主任委员　* 副主任委员
