# Supplementary material for: Electrocardiographic Abnormalities and QTc Interval in Patients Undergoing Hemodialysis
Source: PLoS One. 2016 May 12;11(5):e0155445. doi: 10.1371/journal.pone.0155445 (PMC4865146; doi:10.1371/journal.pone.0155445)
Supplement: S2 File — PLOSOne_Clinical_Studies_Checklist. (DOCX) [file pone.0155445.s002.docx]

***PLOS ONE* Clinical Studies Checklist**

***PLOS ONE* manuscript number: ___**PONE-D-15-46434**____________________**

1. Please ensure that the author list and Corresponding Author entered in Editorial Manager match the author list and Corresponding Author in your manuscript file.

_√__ Completed

1. Please provide an email address for at least one author—preferably the corresponding author—that is hosted by a hospital or university (e.g. authorname@universityname.edu). If no authors have emails hosted by their affiliated university or hospital, state this clearly.

| [ding.xiaoqiang@zs-hospital.sh.cn](mailto:ding.xiaoqiang@zs-hospital.sh.cn)  [nie.yuxin@zs-hospital.sh.cn](mailto:nie.yuxin@zs-hospital.sh.cn) |
| --- |

| **Complete #3-7 if your study involved any human participants or human subjects’ data.**  **These questions should be addressed for prospective and retrospective studies.** | |
| --- | --- |
| 3. | If you did not have ethics approval, please explain why you felt this was not necessary.   \|  \| \| --- \| |
| 4. | Please upload the letter of approval from your ethics committee as file type “Other”.   - If you obtained multiple, sequential approvals for this project, please submit all approval documents that apply to the work reported in this manuscript. - If the approval letter is in another language, please include an English translation.   _√__ Uploaded ___ N/A |
| 5. | If your study involved human participants, please report in the Methods section when participants were recruited to the study.  _√__ Completed ___ N/A |
| 6. | If you are reporting a retrospective study, please report in the Methods section the dates of inclusion for human subjects’ data collected, and specify whether authors had access to identifying information during or after data collection.  _√__ Completed ___ N/A |
| 7. | If you are reporting an observational study, please complete and upload the relevant STROBE checklist (<http://www.strobe-statement.org/index.php?id=available-checklists>) as a Supporting Information file, and update your manuscript to include the requested information.  ___ Completed __√_ N/A |
